# Towards a speech-based digital biomarker for cognitive impairment: speech as a proxy for cognitive assessment

**DOI:** 10.1038/s41746-026-02360-8

**Published:** 2026-01-31

**Authors:** Jonathan Heitz, Ines M. Engler, Nicolas Langer

**Affiliations:** 1 Department of Psychology, Methods of Plasticity Research, University of Zurich, Zurich, Switzerland; 2 Language & Medicine Competence Centre, University of Zurich, Zurich, Switzerland; 3 Neuroscience Center Zurich (ZNZ), Zurich, Switzerland

**Keywords:** Dementia, Alzheimer's disease, Cognitive ageing, Cognitive neuroscience, Machine learning, Psychology

## Abstract

With the growing prevalence of cognitive decline in ageing populations, accessible and scalable screening tools are essential for early intervention. This study investigated the potential of automated speech analysis as a proxy for cognitive assessment in 1003 older adults. Employing machine learning regression models, we demonstrated that linguistic and acoustic features extracted from spontaneous speech quadrupled performance compared to models using demographic information alone, when predicting cognitive domain scores. We then trained a binary classifier to identify individuals performing below normative thresholds (ROC-AUC up to 0.81), illustrating possible applications such as large-scale screening for cognitive impairment and improved participant selection for clinical trials. Finally, we evaluated our approach on an independent clinical dataset of Alzheimer’s disease (AD) patients and controls, demonstrating its generalizability. These findings highlight the clinical feasibility of speech analysis as a low-cost, non-intrusive digital biomarker for cognitive monitoring and screening.

## Introduction

Cognitive decline is a common feature of aging, but its severity and onset vary widely, ranging from mild alterations associated with normative aging to pronounced impairments caused by neurodegenerative diseases such as Alzheimer’s disease (AD)^1^.

With the global population aging rapidly, the prevalence of dementia and other causes of pathological cognitive decline is rising, creating a substantial societal and economic burden^2^. Although biological processes begin decades before symptoms appear, the clinical diagnosis is often delayed by several years, typically occurring only after apparent cognitive impairment affects daily functioning^3^. The effectiveness of disease management and pharmaceutical interventions, including recently approved antibody treatments that slow cognitive decline^4^, decreases with the progression of the disease. Therefore, the therapeutic benefits of these treatments must be initiated as early as possible, and the ability to identify at-risk individuals before symptom onset is crucial.

Current diagnostic procedures of disorders associated with cognitive decline rely on neuropsychological assessment^5^, which typically involves in-person, paper-based tests administered by trained clinicians. These assessments are time-consuming, burdensome for patients, and unsuitable for frequent retesting or large-scale screening. In response, remote, digital, and self-administered cognitive testing approaches have gained traction, a trend accelerated by the COVID-19 pandemic^6^.

In parallel, a growing body of research has highlighted the potential of spontaneous speech analysis as a valuable marker for early detection of cognitive decline. Language-related impairments are among the earliest clinical features of dementia^7,8^, and specific speech patterns have been linked to distinct dementia subtypes^9^. Using speech as a biomarker to shed light on cognitive functioning offers multiple advantages: Speech is easy to collect, low-cost, non-intrusive, and can be captured remotely and repeatedly, making it an attractive candidate for scalable monitoring. Speech characteristics associated with cognition have also shown favorable psychometric properties, such as high test–retest reliability^10,11^.

Previous research has largely focused on using speech to distinguish patients (e.g. MCI, AD, or Parkinson’s disease) from healthy controls (for a review of recent approaches, we refer the reader to Parsapoor^12^ and Filiou et al.^13^). While promising, these studies are often limited by small sample sizes and a focus on moderate to severe AD patients^13^, whose symptoms are apparent in everyday life, thus limiting their utility for early detection.

In contrast to diagnostic classification approaches, predicting cognitive functioning on a continuous scale offers a path towards preclinical speech biomarkers that better reflect the gradual nature of cognitive decline. Such digital biomarkers could support longitudinal cognitive monitoring, trigger alerts in cases of decline, aid large-scale screening to identify at-risk individuals justifying further diagnostic assessments, or improve AD clinical trial recruitment (e.g., enrichment strategies).

Several studies have explored associations between spontaneous speech and global continuous cognitive performance scores: Lindsay et al.^14^ and Ostrand and Gunstad^15^ conducted two correlation analyses, finding significant correlations between linguistic features of spontaneous speech and (Modified) Mini Mental State Examination (MMSE) scores. Agbavor and Liang^16^ predicted MMSE scores using text embeddings derived from modern large language models. Wisler et al.^17^ predicted Montreal Cognitive Assessment (MoCA) scores given measures extracted from recordings of conversational speech from older individuals, although they were able to explain only a small amount of variance in the scores. However, the target cognitive summary scores (MMSE, MoCA) suffer from ceiling effects, particularly in non-clinical populations, and cannot distinguish declines in different cognitive domains. In addition, relatively small sample sizes and the lack of out-of-sample data for evaluation raise concerns about overfitting and the generalizability of these studies.

A related line of research has examined associations between speech features extracted during the administration of neuropsychological tests and performance on individual cognitive tasks (e.g., Thomas et al.^18^ and Ding et al.^19^). While these studies offer insight into the cognitive relevance of speech, their practical utility for early detection is limited. Speech recorded in structured test settings may not generalize well to more naturalistic or unsupervised environments. More importantly, such approaches still require formal neuropsychological testing, thereby undermining the goal of scalable, low-burden screening.

In this study, we address these gaps by investigating whether spontaneous speech analysis can serve as a proxy for cognitive functioning across the cognitive domains of *language*, *executive function*, *memory*, and *speed*. To the best of our knowledge, this is the first study to explore the link between cognitive dimensions and spontaneous speech, providing novel insights into the association of language and cognition.

To this end, we collected a large dataset of spontaneous speech samples and cognitive test results from 1003 English-speaking older individuals. The cognitive tests reflect standard tools commonly used in clinical diagnostics.

We automatically extracted a broad set of linguistic and acoustic features from participants’ speech using natural language processing and speech analysis techniques, and trained predictive machine learning regression models. This well-established methodology has been repeatedly demonstrated to effectively identify signs of cognitive impairment. To ensure robust and generalizable results, we employed a rigorous experimental setup with stratified train/test splits and out-of-sample evaluation.

In addition, we conducted two complementary analyses. First, as a clinically motivated use case, we developed a classifier to identify individuals whose cognitive performance was significantly below demographic expectations (*cognitive low performers*). This innovative application illustrates the potential of speech analysis to support screening for individuals at potential risk of cognitive impairment. Finally, we applied our models to an independent clinical dataset of AD patients and matched controls to test whether speech-derived predictions reflect meaningful cognitive impairment.

## Results

### Dataset characteristics

The demographic characteristics of the participants included in the dataset are summarized in Table 1, with data presented separately for the Development set and the Holdout Test set. Statistical analysis revealed that among the demographic variables, only self-reported socioeconomic status significantly differed between the Development and Holdout Test sets.

**Table 1:** Dataset characteristics on the Development set and Holdout test set

	Development set	Holdout test set	*p*-value
Total number of participants	788	197	
Age	65.50 ± 4.82	65.42 ± 4.77	0.83
*Gender*			0.59
Female	487 (61.8%)	117 (59.4%)	
Male	301 (38.2%)	80 (40.6%)	
*Country*			0.71
UK	394 (50.0%)	95 (48.2%)	
USA	394 (50.0%)	102 (51.8%)	
*Language*			0.19
British English	390 (49.5%)	95 (48.2%)	
American English	386 (49.0%)	102 (51.8%)	
English (other variety)	12 (1.5%)	0 (0%)	
*Education*			0.25
Bachelor	297 (37.7%)	85 (43.1%)	
High school	209 (26.5%)	45 (22.8%)	
Master	127 (16.1%)	30 (15.2%)	
Vocational	89 (11.3%)	26 (13.2%)	
PhD	48 (6.1%)	5 (2.5%)	
Less than high school	18 (2.3%)	6 (3.0%)	
*(Summary)* High education	472 (59.9%)	120 (60.9%)	
*(Summary)* Low education	316 (40.1%)	77 (39.1%)	
*Ethnicity*			0.35
White	720 (91.4%)	179 (90.9%)	
Black	41 (5.2%)	8 (4.1%)	
Mixed	14 (1.8%)	4 (2.0%)	
Asian	7 (0.9%)	1 (0.5%)	
Other	4 (0.5%)	4 (2.0%)	
Socioeconomic status	5.67 ± 1.63	5.30 ± 1.53	0.004
Picture description statistics *(concatenation of Cookie Theft and Picnic Scene)*			
Transcript length (# words)	337.43 ± 293.53	325.86 ± 136.75	0.59
Audio length (s)	156.91 ± 66.47	155.69 ± 54.97	0.81
*Cognitive scores*			
Language	−0.01 ± 1.01	0.03 ± 0.98	0.66
Executive function	−0.03 ± 1.03	0.12 ± 0.89	0.05
Memory	0.02 ± 1.00	−0.10 ± 1.01	0.13
Speed	0.00 ± 1.01	−0.02 ± 0.95	0.81
Mean cognitive score	−0.00 ± 0.79	0.01 ± 0.73	0.84

We report the mean and standard deviation for continuous variables. *p*-values refer to a chi-squared test^66^ (for categorical variables) or a Student’s *t*-test^67^ (for continuous variables). The data split into Development set and Holdout test set and their role in model evaluation are described in section “Predicting cognitive performance from spontaneous speech features”. *Upper part*: Demographic information. *Lower part***:** Statistics on the picture description spontaneous speech task and the composite cognitive score distribution.

The spontaneous speech tasks generated recordings with an average of 73 s and 148 transcription words (Cookie Theft picture description), 87 s and 186 words (Picnic Scene picture description), and 92 s and 186 words (journaling task). In our main analysis, we utilized the concatenation of the Cookie Theft and Picnic Scene picture description tasks, the statistics of which are presented in Table 1. The audio recordings’ mean signal-to-noise ratio was 35 dB, indicating high-quality recordings containing clean speech with barely audible noise.

To validate the quality of our transcriptions, we conducted an error analysis comparing automatic and manual transcriptions for a randomly selected subset of 40 Cookie Theft picture descriptions. This analysis yielded an average Word Error Rate of 0.073, demonstrating the high accuracy of the automated transcription process. Furthermore, the automatically generated scores of the structured language tasks (Boston Naming task, phonetic and semantic fluency) exhibit high agreement to manual human scoring, with intra-class correlation (ICC) above 0.97 and mean absolute errors below 1. Detailed results are presented in Supplementary Table 1.

**Fig. 1:**
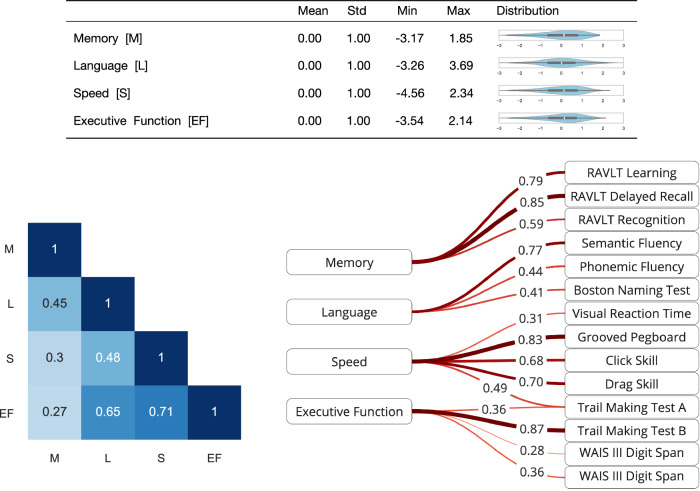
Overview of the composite cognitive scores. *Top*: Distribution statistics for composite cognitive scores. Due to score standardization the mean and standard deviation are set to 0 and 1 for all composite scores. *Bottom left*: Correlation between composite cognitive scores. *Bottom right*: A path diagram of the CFA model displaying the assignment and factor loadings of individual test items for each cognitive domain. Line thickness and color saturation indicate the magnitude of factor loadings. The cross-loading of Trail Making Test A was the result of model refinement based on modification indices. RAVLT: Rey auditory verbal learning test. WAIS: Wechsler adult intelligence scale.

We employed confirmatory factor analysis (CFA) to derive four cognitive composite scores (representing the cognitive domains *language*, *executive function*, *speed*, and *memory*) from the individual cognitive test scores, statistics of which are presented in Supplementary Table 2. Our CFA factor model demonstrated strong cohesion and provided a robust fit to the data (robust CFI: 0.94, TLI: 0.92, RMSEA: 0.06, details provided in Supplementary Table 3). The resulting factor loading structure is presented in Fig. 1. We observe that the composite scores are correlated to each other, aligning with the theory that cognitive domains are not independent^20^.

### Predicting cognitive performance from spontaneous speech features

**Fig. 2:**
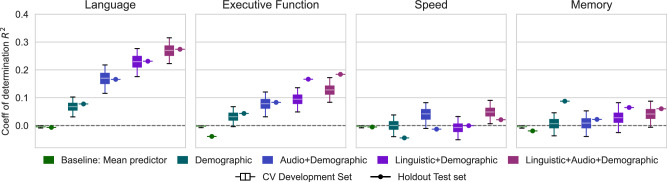
Visual representation of SVR results for the prediction of cognitive performance. The coefficient of determination (*R*^2^) is visualized for different cognitive composite scores and feature sets. Box plot whiskers represent the 95% bootstrap confidence intervals of the results on the Development set. These results correspond to the tabular representation in Table 2.

We trained support vector regression (SVR) models to predict cognitive performance for each cognitive domain, given features extracted from participants’ picture description recordings. These linguistic and acoustic features successfully captured important speech characteristics, quadrupling predictive performance compared to the use of demographic information alone (Table 2, Fig. 2). More specifically, the most accurate predictions derived from spontaneous speech features were produced for the cognitive composite score *language* (*R*^2^ = 0.27), followed by *executive function* (*R*^2^ = 0.13), and *speed* (*R*^2^ = 0.05). We also observed a trend of linguistic features outperforming acoustic features, and their combination consistently produced the best results. The cognitive domain *memory* (*R*^2^ = 0.04) could not be explained by speech measures. Additional performance metrics (Spearman correlation coefficient, mean absolute error and detailed statistical results are reported in Supplementary Tables 4 and 5, respectively.

**Table 2:** SVR results for cognitive composite scores *executive function*, *language*, *memory*, and *speed*

Coefficient of determination *R*^2^	Language		Executive function	
	CV Developm set	Holdout test	CV Developm set	Holdout Test
Baseline: Random	−1.08 [−1.32, −0.87]	−0.81	−1.10 [−1.32, −0.90]	*t*−1.30
Baseline: Mean predictor	−0.00 [−0.00, −0.00]	−0.00	−0.00 [−0.00, 0.00]	−0.03
Demographic features	0.07 [0.03, 0.10]	0.08	0.03 [−0.00, 0.07]	0.05
Audio + Demographic features	0.17 [0.12, 0.22]	0.17	0.08 [0.03, 0.12]	0.09
Linguistic + Demographic features	0.23 [0.18, 0.28]	0.24	0.10 [0.05, 0.14]	0.18
Linguistic + Audio + Demographic features	0.27 [0.22, 0.32]	0.29	0.13 [0.09, 0.18]	0.20

Coefficient of determination *R*^2^	Speed		Memory	
Baseline: Random	−0.85 [−1.07, −0.69]	−1.03	−1.10 [−1.35, −0.91]	−1.01
Baseline: Mean predictor	−0.00 [−0.00, −0.00]	−0.00	−0.00 [−0.00, −0.00]	−0.01
Demographic features	−0.00 [−0.04, 0.04]	−0.04	0.01 [−0.04, 0.05]	0.09
Audio+Demographic features	0.04 [−0.02, 0.08]	−0.01	0.01 [−0.04, 0.06]	0.03
Linguistic + Demographic features	−0.01 [−0.05, 0.03]	0.00	0.03 [−0.02, 0.09]	0.07
Linguistic + Audio + Demographic features	0.05 [0.01, 0.09]	0.03	0.04 [−0.00, 0.09]	0.07

We report the coefficient of determination (*R*^2^) using different combinations of demographic, acoustic, and linguistic features. In addition, we report results of two dummy baseline regressors, predicting the training set’s mean or a random training target. Results for 10-fold cross-validation on the Development set (*CV Developm set*) are accompanied by 95% bootstrap confidence intervals.

**Fig. 3:**
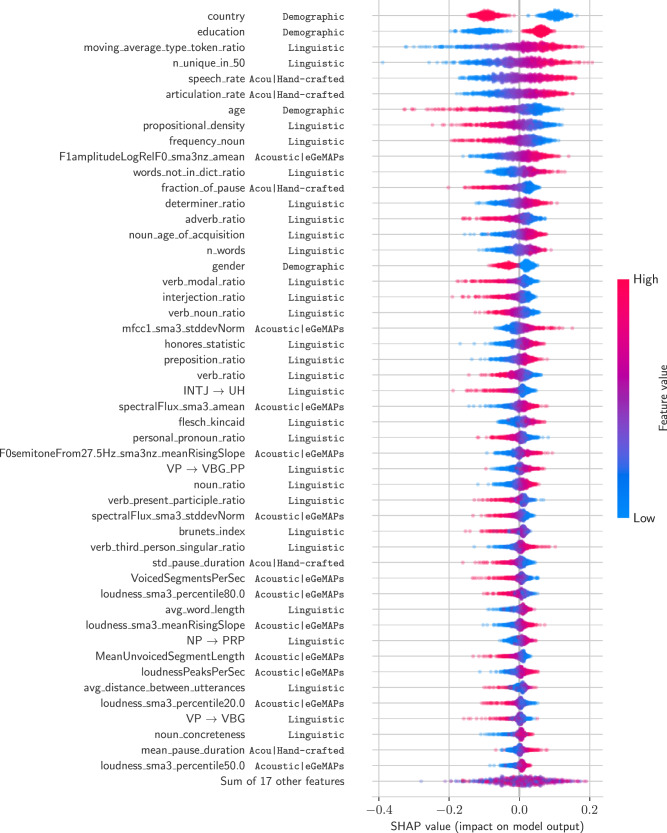
Visualization of SHAP feature importances in our main SVR regression model, predicting the composite cognitive score *language.* Each data point represents a test participant’s prediction in cross-validation on the Development set. Positive/negative SHAP values indicate a higher/lower predicted *language* score, whereas red and blue refer to higher/lower feature values. For example, a higher age (i.e., red dot) indicates a lower predicted *language* score. Features are ordered by their mean absolute SHAP value, a global metric of feature importance. Results for the composite cognitive scores *executive function*, *speed*, and *memory* are provided in Supplementary Fig. 1.

The subsequent feature importance analysis for the *language* score predictions demonstrated that all feature groups (demographic, linguistic, and acoustic features) contribute to predicting the composite cognitive score (Fig. 3). The demographic variable capturing the country of origin (USA vs. UK) and the educational background contributed most strongly to the individual predictions on average. Among linguistic features, measures of lexical diversity (*moving_average_type_token_ratio* and *n_unique_in_50*), the frequency of the uttered nouns (*frequency_noun*), and measures based on the ratio of specific parts of speech (*propositional_density*, *determiner_ratio*, *adverb_ratio*) emerged as strong predictors. For acoustic features, variables relating to speaking speed (the *speech_rate* and *articulation_rate*), pause duration (*fraction_of_pause*), and voice frequencies (*F1amplitudeLogRelF0_smxa3nz_amean*, the relative energy of formant 1 compared to pitch) demonstrated the highest relevance. For the cognitive domains *executive function*, *memory*, and *speed*, similar features proved important, the details of which are presented in Supplementary Fig. 1.

Our control analyses revealed no consistent bias in model performance across age groups, educational levels, genders, or countries (detailed results in Supplementary Fig. 2), indicating that the model generalizes well across demographic subgroups.

We also compared model performance across different spontaneous speech tasks. The use of the concatenated picture descriptions (our main analysis) yielded slightly better predictive performance than using individual picture descriptions or the journaling task alone (detailed results in Supplementary Table 6). However, the overall findings remained consistent: models performed best in *language* and *executive function* domains, and incorporating speech features consistently improved prediction beyond demographic variables, regardless of the speech elicitation task. Furthermore, results from the Random Forest Regression (Supplementary Table 7) aligned with those from SVR but did not yield improved predictive performance, and the addition of *socioeconomic status* to the set of demographic variables did not prove advantageous (Supplementary Table 8).

### Detection of cognitive low performers

**Fig. 4:**
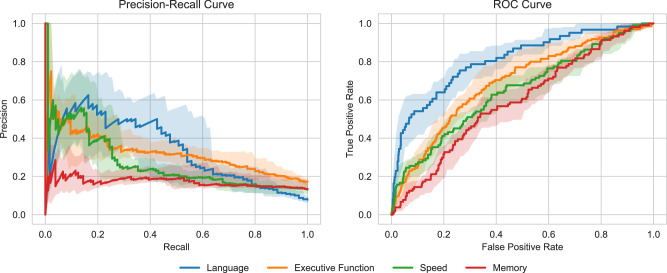
SVM classification results for the detection of cognitive low performers. Receiver operating characteristic (ROC) curve and precision-recall (PR) curve on the Development set, with shaded areas representing 95% bootstrap confidence intervals.

The clinical utility of speech-based biomarkers lies primarily in the identification of individuals with a cognitive performance below expected norms, as this could be the result of pathological processes. Considering our datasets’s sample size and age range, it is plausible that some participants were already experiencing early cognitive decline. Motivated by this observation, we identified *cognitive low performers* for each cognitive domain as participants with *z*-scores below −1.96, indicating significant negative deviation from the normative sample. The number of *cognitive low performers* varies by cognitive domain, ranging from 7.8% to 17.2% of the data (Table 3). Our trained SVM classifier achieved an ROC-AUC of 0.81 and a PR-AUC of 0.33 in identifying low performers in the *language* domain (Fig. 4, Table 3). The classifier demonstrated lower discriminatory power in identifying low performers in the *executive function* (ROC-AUC: 0.69, PR-AUC: 0.32) and *speed* (ROC-AUC: 0.65, PR-AUC: 0.26) domains, while still performing better than chance. The classifier’s identification of low performers in the *memory* domain (ROC-AUC: 0.59, PR-AUC: 0.17) was only slightly better than the no-skill baselines (PR-AUC = 0.13, the prevalence of the positive class, and ROC-AUC = 0.5, the random baseline). These results are consistent with our main findings (Fig. 2), where regression performance was highest for the *language* domain, followed by *executive function*, *speed*, and *memory*. Results of an alternative Random Forest classification model (Supplementary Table 9) aligned with those from the SVM without improving predictive performance.

**Table 3:** Descriptive sample statistics and SVM classification results for the detection of cognitive low performers: ROC and PR area under the curves for 10-fold cross-validation on the Development set (CV Dev) and on the Holdout Test set (Holdout Test)

	# Samples		# Cognitive Low Performers		PR-AUC		ROC-AUC	
	CV Dev	Holdout test	CV Dev	Holdout test	CV Dev	Holdout test	CV Dev	Holdout test
Language	783	196	61 (7.8%)	8 (4.1%)	0.33 [0.26–0.44]	0.09	0.81 [0.74–0.86]	0.75
Executive function	783	196	135 (17.2%)	26 (13.3%)	0.32 [0.28–0.37]	0.23	0.69 [0.66–0.72]	0.61
Speed	783	196	102 (13.0%)	23 (11.7%)	0.26 [0.24–0.35]	0.21	0.65 [0.61–0.70]	0.69
Memory	783	196	104 (13.3%)	33 (16.8%)	0.17 [0.14–0.18]	0.19	0.59 [0.55–0.63]	0.58

CV Development set results are accompanied by 95% bootstrap confidence intervals. The linear norms defining the assignment as cognitive low performers are given in the Supplementary Fig. 4.

### Evaluation on clinical dataset of AD patients and controls

**Fig. 5:**
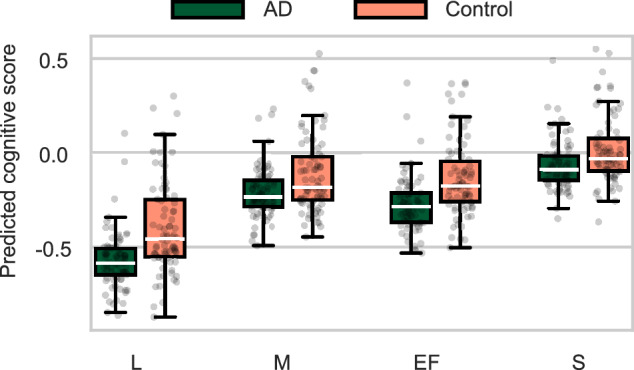
Predicted scores for AD patients and healthy controls. Box plots depicting the distribution of scores are overlaid by a scatter plot of individual predictions.

We applied the predictive models of our main analysis, which are trained on our primary dataset, to generate predicted composite cognitive scores for participants in an independent clinical dataset containing picture description recordings of AD patients and healthy controls. In line with our expectations, AD patients showed significantly lower predicted scores for all cognitive domains (Fig. 5, Table 4). Effect sizes were medium to large for *memory* (*d* = 0.60) and *speed* (*d* = 0.51), and large to very large for *language* (*d* = 0.90) and *executive function* (*d* = 0.80). We want to highlight that the difference between these effect sizes should not be interpreted as the extent to which different cognitive domains are affected by AD. Instead, we hypothesize that it aligns with the amount of information about these cognitive domains contained in our predictive models (as presented in our main regression results in Fig. 2).

**Table 4:** Effect size (Cohen’s *d*) as well as *t*-statistic and *p*-value of a Student’s *t*-test for the difference in mean between predicted scores for AD patients and healthy controls on the ADReSS dataset

	Effect size	*t*-statistic	*p*-value
Language [L]	0.90	5.6	1.4e−07
Memory [M]	0.60	3.7	2.8e−04
Executive Function [EF]	0.80	5.0	1.7e−06
Speed [S]	0.51	3.2	1.7e−03

These results demonstrate the clinical significance of our approach, highlighting that scores representing the performance of cognitive domains derived from spontaneous speech features on a sample of healthy older participants generalize to a clinical AD population.

## Discussion

This study examined whether spontaneous speech can predict cognitive functioning in older adults. Using SVR with out-of-sample evaluation, we demonstrated that spontaneous speech features contain significant information about cognitive performance, explaining up to 27% of the variance in domain scores. Subsequently, we leveraged these speech features in a binary classification task, identifying individuals with cognitive performance below what can be expected given their demographic information. This could support clinical use cases such as screening for cognitive impairment and improved participant selection for clinical trial recruitment. Finally, we evaluated our approach on clinical data, demonstrating that the regression model trained on our non-clinical dataset of older participants generalized without any modifications to an independent dataset of AD patients and healthy controls. The predicted cognitive scores were significantly lower for AD patients than for controls across all cognitive domains, demonstrating both the model’s clinical validity and generalizability. In the following sections, we will discuss our findings in more detail.

Most prior research has largely focused on distinguishing healthy controls and clinical groups affected by pathological cognitive decline. The differences between these groups are often obvious and clearly extend into speech production, whereas speech-based screening would be relevant primarily in populations with at most slight cognitive impairment. This study demonstrated that variability in cognitive function is indeed reflected in language use in such a population of older adults. This finding is noteworthy because spontaneous speech, unlike formal cognitive tasks, imposes a relatively low cognitive load^21^ and is generally effortless in everyday contexts. The fact that cognitive differences are still detectable suggests a robust link between language and underlying cognitive capacity.

Importantly, our results demonstrate that predictive performance increased dramatically when speech features were added to a model using only demographic information. In *language* and *executive function* domains, the explained variance more than quadrupled, underscoring the substantial added value of spontaneous speech. This improvement was driven by both linguistic and acoustic features, with linguistic features consistently showing stronger contributions, confirming previous findings^22,23^.

By relying on explicit, human-interpretable features and leveraging SHAP values, we were able to identify which aspects of speech were most informative. This level of interpretability is essential for clinical adoption, as explainability is a central requirement for ethical and legally compliant use of AI in healthcare^24^. In addition, our approach is computationally efficient: feature extraction and inference could be run locally on a user’s device, ensuring privacy of sensitive information. Recently, deep learning architectures and fine-tuning of pre-trained language models (such as BERT or GPT) have gained considerable traction^25^, as they have shown high predictive accuracy in several benchmark tasks and real-world applications. However, they offer limited interpretability and typically require significant computational resources, which often necessitate processing data on external servers, introducing additional privacy and security risks that may hinder clinical implementation. In addition, the performance of deep learning and interpretable feature-based models is often similar and context-dependent. One of the few rigorous statistical comparisons showed a slight but non-significant advantage of fine-tuned BERT compared to feature-based approaches^26^, a finding supported by Roshanzamir et al.^27^. In contrast, Kumar et al.^28^ found that feature-based models outperformed a deep learning approach. In our own prior work, fine-tuned BERT outperformed feature-based models when using manual transcripts, but not when using automatic speech recognition output^29^, highlighting the sensitivity of results to the exact pipeline and data quality. More recently, we found that incorporating GPT-derived features in a feature-based pipeline yielded the best results, while fine-tuned GPT alone performed suboptimally^30^.

Our feature importance analysis revealed that features reflecting semantic specificity were the most predictive of cognitive functioning. This confirms prior research^15^ and is measured by features such as the type-token ratio, the fraction of unique words, the lexical frequency and age of acquisition of uttered nouns, and ratios of verbs, prepositions, and determiners. On the acoustic level, the fluency of speech production, including speaking rate and the use of pauses are characteristics informative of cognitive functioning, which are findings in line with previous research^31,32^. Surprisingly, the country of origin (USA vs. UK) emerged as an important feature, particularly for the *language* cognitive domain. A supplementary analysis revealed that composite cognitive scores were significantly higher among UK participants compared to US participants (Supplementary Table 13). Because these differences remained significant after accounting for demographic variables (Supplementary Table 14), we tested whether systematic differences in the automated scoring of the language tasks might underlie this effect. However, when compared with manual scoring, the automated scoring proved highly reliable (ICC = 0.85−0.98), and the discrepancy between manual and automatic scores was small (error < 1 point) and statistically indistinguishable between countries (Supplementary Table 15). Furthermore, the UK participants also showed higher scores in non-language domains (e.g., *speed* and *executive function*) that are not influenced by automated scoring. Together, these findings suggest that the observed country effect likely reflects an unobserved selection bias within the Prolific participant pool.

Crucially, the predictive value of speech features varied across cognitive domains, with the strongest performance observed for the *language* composite score. This score is derived from speech-based tasks that share many influencing factors with spontaneous speech production. These findings are encouraging, as these underlying tests, like semantic fluency, are known to be sensitive to the earliest signs of cognitive impairment caused by various variants of dementia, including AD^33^ and semantic dementia^34^. Spontaneous speech also successfully predicted *executive function*, likely due to overlapping cognitive demands such as working memory and planning, which are processes that also support fluent speech^35^. This is supported by the fact that the Trail Making Test B, known to engage in these processes^36^, was the primary contributor to the *executive function* composite score. While still explaining some variation, the results were less convincing for our composite score *speed*. Although some of our speech features (e.g., *speech_rate*) are speed-related, we speculate that the modest prediction performance is caused by the cognitive *speed* score being primarily driven by tasks that measure psychomotor speed, which likely has limited relevance on speech production. Our speech-based measures, on the other hand, presumably capture a different subset of the broader *speed* domain, which is multifaceted and encompasses distinct types of cognitive and motor speed^37^. Finally, our speech features were unable to capture meaningful information about the *memory* cognitive domain. We hypothesize that this lack of predictive power was due to a mismatch between our speech elicitation tasks and this cognitive domain score. The *memory* score is based on Rey Auditory Verbal Learning Test results and thus captures verbal memory and learning^38^, which are likely not required in the picture description task. The alternative journaling task requires some episodic memory rather than verbal memory, and results remain poor (Supplementary Table 6). A task asking participants to retell a story or describe a previously presented picture from memory should be explored in future studies and might enable a more accurate representation of this cognitive domain. Overall, these differences between cognitive domains highlight the importance of studying cognition on a more granular level than what cognitive summary scores such as MMSE or MoCA can offer, and explain why research by Wisler et al.^17^ reported a limited predictability of MoCA scores from measures of spontaneous speech. Accurately capturing cognitive decline in two clinically relevant domains (*language* and *executive function*) is sufficient to develop a speech-based biomarker for various screening applications.

While most of the cognitive variability observed in our sample is within the range of healthy aging, one key application of speech-based digital biomarkers lies in identifying individuals whose cognitive performance falls below expected norms, as this could potentially reflect prodromal stages of dementia. Given the age distribution of our participants, it is plausible that some were already experiencing early cognitive decline. To explore this, we developed a classifier to detect *cognitive low performers*, defined as individuals whose scores fell significantly below expectations based on demographic characteristics. The model achieved encouraging results, with an area under the ROC curve (AUROC) of up to 0.81. A tool capable of accurately and automatically identifying *cognitive low performers* from spontaneous speech could serve as a valuable and easily deployable low-cost screening method to flag individuals for further clinical assessment. Early detection is critical for timely intervention and may enable earlier access to treatment or support. Beyond clinical screening, this approach has important implications for clinical trial recruitment. Many AD trials rely on cognitive thresholds to enrich samples with individuals likely to progress to AD. Recent work (e.g., Tam et al.^39^) has shown that predictive biomarkers, such as MRI-based models can reduce trial costs by improving participant selection. Speech analysis represents a promising, far more scalable and cost-effective alternative. This is supported by recent studies by Petti and Korhonen^40^ and Mueller et al.^7^, who have demonstrated that subtle speech changes may appear years before clinical AD diagnosis. Similarly, Favaro et al.^41^ showed the potential of speech-based models in identifying prodromal Parkinson’s disease. Our results contribute to this growing body of evidence. As shown in Fig. 4, the precision-recall curves illustrate that speech from a simple picture description task can identify a subgroup with a high prevalence of cognitive low performers, making them promising candidates for clinical trial enrichment.

To be clinically useful, our approach should also generalize to more severe presentations of cognitive impairments, such as those seen in AD. Therefore, we evaluated the SVR model, trained on our non-clinical primary dataset, to predict cognitive scores from the recordings contained in the ADReSS dataset, which includes AD patients and healthy controls. As expected, the predicted scores for AD patients were significantly lower than those of controls across all domains, with large effect sizes (Table 4). This is remarkable, as our models did not see any AD patients in their training data, and the distributional shift between the datasets is substantial: The ADReSS dataset was recorded in the 1980s, contains high levels of background noise affecting speech intelligibility, and exhibits a distinct demographic profile. This result highlights the robustness of our speech-based approach, a critical requirement for real-world deployment, where control over microphone hardware and background noise is limited.

As with any study, ours also has limitations. First, its cross-sectional design prevents conclusions about changes over time. Longitudinal studies are needed to track intraindividual changes in speech and examine their relationship to cognitive decline, particularly to identify individuals experiencing pathological decline. Second, our sample consisted of native English speakers from the USA and the UK. While this supports internal consistency, it may limit generalizability to other dialects or non-native speakers. More diverse datasets could improve generalizability, although this would also increase variability in spontaneous speech due to non-cognitive factors, which makes effects harder to detect in a cross-sectional design. This further highlights the need for longitudinal data. While some prior studies indicated that speech-based approaches can generalize across languages (e.g., refs. ^42,43^), further work is needed to evaluate and adapt our approach to languages other than English. Third, remote data collection via Prolific is influenced by a myriad of unobserved factors, such as hardware, distractions, background activity, or interruptions. While data quality checks ensured compliance with task instructions, and a high signal-to-noise ratio and low word error rate suggest good audio quality, the nature of our data collection can influence both cognitive test results and features extracted from the speech recordings. A more controlled setting would likely produce a clearer signal. On the other hand, this setup allowed us to collect an unusually large sample of participants, and we believe it to be more ecologically valid, thus likely generalizing better to practical data collection in potential clinical applications. Fourth, we followed a well-established methodological approach of linguistic and acoustic feature extraction in combination with general-purpose machine learning models. This approach has been successfully employed by previous research and offers advantages with respect to interpretability. However, alternative approaches based on fine-tuning or deep learning could uncover more subtle signs of cognitive impairment and should be further explored in future research. Fifth, the present study recruited older adults, most of whom are likely to be cognitively healthy. However, as cognitive status was not determined by a clinical specialist, it is possible that some participants were affected by undiagnosed cognitive impairment. Future studies should formally assess participants’ cognitive status and explicitly include individuals with mild cognitive impairment or dementia, to further study which speech characteristics are affected by pathological cognitive decline. The evaluation of the proposed approach on clinical data supports its potential to generalize to clinical populations, although full clinical validation will require dedicated prospective studies in diagnostic settings. Lastly, the clinical implementation of speech-based screening systems raises some ethical risks, particularly relating to consent (as cognitive decline can affect decision-making capabilities), privacy (as speech samples can identify individuals and potentially contain sensitive information), and potential distress caused (especially relating to false positives)^44^. Careful implementation in a larger clinical setting is required to mitigate these risks.

This study evaluated the extent to which analysis of spontaneous speech could predict cognitive performance in a large sample of older adults. Our results demonstrated that speech characteristics could explain cognitive domain scores for *language* and *executive function* to a significant degree, but were less predictive for *speed* and *memory*. Our approach was able to identify cognitive low performers, making it suitable for a range of clinical applications, and was shown to generalize to a clinical population with AD. These results highlight the potential of language as an efficient, robust, inexpensive, and non-intrusive digital biomarker of cognition, with applications in cognitive monitoring, detection of cognitive impairment, and clinical trial recruitment. Future research should collect large longitudinal datasets of spontaneous speech and cognitive testing.

## Methods

### Participant recruitment

Participant recruitment took place between April and July 2024 on Prolific, an online crowdsourcing platform with more than 200,000 verified global users. Research has shown high data quality of Prolific users’ responses^45^. Study participation was restricted to older native English speakers from the USA or UK to minimize speech variations caused by cultural or linguistic background, by applying the following demographic inclusion criteria: (a) age 60 or older, (b) first language English, (c) USA or UK nationality, (d) current country of residence in the USA or UK, and (e) most time spent before turning 18 in USA or UK. In addition, a desktop or laptop computer with internet and microphone access was required to participate.

A total of 1003 participants successfully completed the full online study and were included in the final analysis. Additional individuals began the study but were excluded due to incomplete participation, technical issues, or unresolved data quality concerns (see section “Data preprocessing” for details).

Successful participation in our online study took ~90 min and was compensated with £13.50, corresponding to £9.00 per hour, which is considered fair pay and recommended by Prolific. The study was approved by the ethics committee of the Faculty of Arts and Social Sciences at the University of Zurich (Number 23.08.03). Participants consented to the use of their data for the purposes of this study. Participant speech was recorded using the computer’s built-in microphone during both the spontaneous speech tasks and the standardized language tasks. Participant data and recorded audio files were stored on a secure web server running the study.

### Data collection

Our study consisted of four parts: a demographic survey, three tasks designed to elicit spontaneous speech, an online neuropsychological test battery, and several standardized language tasks.

Participants provided *demographic information*, including age (continuous), gender (male or female), country of residence (USA or UK), socioeconomic status (1–10, according to the MacArthur Scale of Subjective Social Status^46^), and education. Education was binarized based on the International Standard Classification of Education (ISCED)^47^, with low-education corresponding to ISCED 0–4 and high-education corresponding to ISCED 5–8.

Participant speech was recorded using the computer’s microphone during three *spontaneous speech tasks*: two picture description tasks and one journaling task. In the picture description tasks, participants were asked to describe everything happening in a black-and-white drawing. The two chosen pictures (The Cookie Theft picture from the Boston Diagnostic Aphasia Examination^48^ and the Picnic Scene picture from the Western Aphasia Battery^49^) have originally been developed to diagnose and differentiate forms of aphasia, and have been used extensively in research analyzing speech and related cognitive deficits in diverse clinical populations, including individuals with dementia^50^. In our journaling task, participants were asked to describe their past week, eliciting speech that is more ecologically valid, but influenced more strongly by individual context. For all three tasks, participants were instructed to provide as many details as possible and to speak for at least one minute, with no upper time limit.

To measure cognitive performance, our study included the Amsterdam Cognition Scan (ACS), a validated neuropsychological test battery covering a range of cognitive domains such as attention, memory, executive function, and processing speed^51^, with included tests listed in Table 5.

**Table 5:** Cognitive tests included in our study: The Amsterdam Cognition Scan battery (top) and the three standardized language tasks (bottom)

Online test	Main outcome measure
Trail making test A and B^68^ (adapted online version) Rey auditory verbal learning test^69^ (adapted online version) Visual reaction time (subtest FePsy)^70^ (adapted online version) Tower of London, Drexel University (TOL-dx)^71^ (adapted online version) Corsi Block-tapping test^72^; (adapted online version) Grooved Pegboard^73^ (adapted online version) WAIS III Digit Span (forward and backward)^74^ (adapted online version) Clicking speed test Mouse dragging speed test	Completion time (A and B) Learning: Total number of correct words (trial 1–5) Recall and recognition: Total number of correct words Mean reaction time Total number of extra moves Completion time Completion time Total number of correctly repeated sequences (forward and backward) Completion time Completion time
Phonemic Fluency ("F" fluency) Semantic Fluency (Category fluency) Boston naming test^52^	Number of valid words Number of valid words Number correctly named objects

Note that ACS uses different names to refer to the tests. For clarity, we use the names of the traditional equivalent test as reported in Feenstra et al.^51^.

Note that the ACS uses different names to refer to the tests. For clarity, we use the names of the traditional equivalent test as reported in Feenstra et al.^51^. While the ACS covers various domains of cognitive functioning, it lacks language-specific tests. For this reason, we included an online version of three *standardized language tasks* widely used in research and in clinical neuropsychological assessments: (1) a *semantic fluency* task, where participants were asked to name as many animals as they could think of within one minute, (2) a *phonemic fluency* task, asking participants to name as many words as possible starting with the letter “F” within one minute, and (3) a shortened version of the *Boston Naming Test*^52^, where participants were shown drawings of 20 objects, which they were asked to name. The included fluency tasks are popular for their quick and easy administration and their sensitivity to impairments in verbal ability and executive control, and are especially popular as cognitive measures in the context of early detection of MCI and AD^53,54^. The Boston Naming Test is a confrontation naming test and among the most frequently administered neuropsychological tests^38^, assessing word retrieval difficulties associated with various neurological conditions.

Since participants were unsupervised and compensated with a fixed amount, there was a danger of participants rushing through the tasks (especially the spontaneous speech tasks) very quickly, affecting the reliability and validity of the collected data. To manage this risk and avoid taking unreliable assessments into account, we checked data for completeness and adherence to the instructions. Spontaneous speech audio recordings with durations of less than the requested one minute were manually checked and considered sufficient if the task was completed adequately. Otherwise, the participants were contacted via Prolific’s messaging system, where they were asked to redo the tasks. Participants who were unable to resolve the issues were removed from the dataset. To quantify the level of background noise, we computed a signal-to-noise ratio of all recordings, based on an automatic segmentation into voice segments (i.e., signal) and pause segments (i.e., noise).

### Data preprocessing

Participant information, recorded audio files, and ACS test results were preprocessed to prepare the data for analysis.

The transcriptions of the speech recordings were generated using automatic speech recognition (ASR). We employed Google Speech ASR (*chirp* model^55^), which has provided excellent-quality transcription in our previous work on spontaneous speech of older participants^29^. The model is available via a Google Cloud API. To assess the quality of the generated transcriptions, we manually transcribed a random subset of 40 Cookie Theft picture description audio files. We then computed the Word Error Rate, a common evaluation metric for automatic speech recognition^56^.

To ensure data quality, we identified and removed outliers in the cognitive scores derived from the ACS, and imputed the resulting missing values. Outliers include unusually long reaction times, likely resulting from participants pausing during tasks. We fitted a multiple linear regression model for each cognitive variable using demographic predictors. Observed ACS scores with absolute standardized *z*-scores > 4 were classified as outliers and replaced with missing values. This procedure identified between 0 (0%) and 35 (3.5%) outliers per test. The regression model parameters are presented in Supplementary Table 10. Missing values were subsequently imputed using a multivariate Round-Robin regression strategy based on Bayesian ridge regression, based on the remaining cognitive scores from the specific subject^57^.

We developed an automatic scoring algorithm for the three standardized language tasks (semantic fluency, phonemic fluency, and Boston naming test). The scoring is based on automatically generated transcripts of the audio recordings and a specialized logic counting valid animal names based on a dictionary (for semantic fluency), counting valid words starting with “F" (for phonemic fluency), and counting correctly named objects (for the Boston naming test). To assess the quality of our scoring algorithm, we manually scored these tasks for 40 participants not used in the development of the algorithm, and computed the algorithm’s mean absolute error and intra-class correlation compared to the manual scores.

To create robust composite scores for cognitive performance, we integrated individual test results from the ACS battery and the three standardized language assessments into four key cognitive domains: *memory*, *executive functioning*, *language*, and *speed*. This approach was informed by Mukherjee et al.^58^, where a panel of experts theoretically mapped a broad range of cognitive tests from multiple studies to the domains of *memory*, *executive functioning*, and *language*, employed confirmatory factor analysis to validate their assignment, and demonstrated biological coherence of AD subgroups derived from these domains. Mukherjee et al.^58^ additional cognitive domain *visuospatial functioning* was excluded due to the absence of respective test items in the ACS battery. We expanded the framework by including *speed* as a fourth domain, recognizing the critical role of processing speed in cognitive aging^59^ and the availability of relevant measures within the ACS battery (*Visual reaction time*, *Grooved Pegboard*, *Clicking speed test*, and *Mouse dragging speed test*). To validate the derived assignment on our data, we employed confirmatory factor analysis (CFA) using R’s lavaan package. We split our data into two stratified halves and used the first half to iteratively refine a factor model, including cross-loadings or error covariances with modification indices (MI) larger than 16, while ensuring that modifications were theoretically justifiable. The improved model was then validated using the second data half. A maximum likelihood estimator was used for model fitting, and the fit for both data halves was assessed through robust root mean square error of approximation (RMSEA), robust comparative fit index (CFI), and robust Tucker–Lewis index (TLI). After establishing a model optimal to our data (lavaan syntax given in Supplementary Fig. 3), we computed the four composite cognitive scores for each participant based on a final fit to the entire dataset. Finally, composite scores were standardized by subtracting the mean and dividing by the standard deviation, ensuring comparability across participants and cognitive domains.

### Speech feature extraction and preprocessing

We applied natural language processing (NLP) and acoustic analysis techniques to automatically extract features from participants’ spontaneous speech recordings. *Linguistic features* were derived from the textual transcripts using NLP methods, while *acoustic features* were directly computed from the raw audio signals. This dual-modal feature extraction approach allowed us to capture a broad spectrum of information, ranging from syntactic and lexical properties of speech to fine-grained acoustic parameters. A detailed overview of all extracted features is provided in Table 6.

**Table 6:** Linguistic and acoustic features were extracted for the spontaneous speech audio recordings and corresponding transcripts

*Feature group*		
	Feature name	Description
*Linguistic features — Lexical*		
	n_words	Number of words in the transcript
	verb_noun_ratio	Ratio of verbs to nouns
	subordinate_coordinate_con-junction_ratio	Ratio of subordinate to coordinate conjunctions
	adverb_ratio	Ratio of adverbs to all words
	noun_ratio	Ratio of nouns to all words
	verb_ratio	Ratio of verbs to all words
	pronoun_ratio	Ratio of pronouns to all words
	personal_pronoun_ratio	Ratio of personal pronouns to all words
	determiner_ratio	Ratio of determiners to all words
	preposition_ratio	Ratio of prepositions to all words
	verb_present_participle_ratio	Ratio of verbs (present participle) to all words
	verb_modal_ratio	Ratio of modal verbs to all words
	verb_third_person_singular_ratio	Ratio of verbs in 3rd person singular to all words
	interjection_ratio	Ratio of interjections (mostly hesitations, such as uh) to all words
	avg_word_length	Average word length (number of letters)
	words_not_in_dict_ratio	Number of words not in an English dictionary
	brunets_index	Brunét’s index^75^, a measure of lexical richness
	honores_statistic	Honoré Statistic^76^, a measure of lexical richness
	moving_average_type_token_ratio	The moving average type-token ratio^77^ with a window size of 20 words
	n_unique_in_50	Average number of unique words in a 50-word sample^17^
	propositional_density	Propositional density^78^: Ratio of verbs, adjectives, adverbs, prepositions, and conjunctions to all words
	content_density	Content density^78^: Ratio of nouns, verbs, adjectives, and adverbs to all words
	frequency_noun	Mean word frequency of nouns^79^
	age_of_acquisition_noun	Mean age of acquisition of nouns^80^
	familiarity_noun	Mean word familiarity of nouns^80^
	ambiguity_noun	Mean ambiguity of nouns^81^
	noun_concreteness	Mean concreteness of words^82^
*Linguistic features—Syntactic*		
	NP → PRP	Count of production rules based on constituency parsing
	ROOT → FRAG	Count of production rules based on constituency parsing
	VP → VBG	Count of production rules based on constituency parsing
	VP → VBG_PP	Count of production rules based on constituency parsing
	VP → VBD_NP	Count of production rules based on constituency parsing
	INTJ → UH	Count of production rules based on constituency parsing
	NP_ratio	Ratio of NP constituents
	VP_ratio	Ratio of VP constituents
	avg_n_words_in_NP	Average number of words in a noun phrase
	flesch_kincaid	Flesch-Kincaid Grade Level Formula Kincaid et al.^83^, a measure of readability.
*Linguistic features — Semantic*		
	avg_distance_between_utterances	The average cosine distance between two sentences’ TF-IDF vectors, based on Masrani et al.^84^’s implementation
	prop_utterance_dist_below_05	Proportion of sentence pairs with cosine distance < = 0.5, based on Masrani et al.^84^.
*Acoustic features ∣ Hand-crafted*		
	audio_length	Audio length in seconds
	fraction_of_pause	Fraction of pause segments over audio length
	mean_duration	Mean pause duration
	std_duration	Standard deviation of pause durations
	speech_rate	Number of spoken phonemes divided by the total audio length
	articulation_rate	Number of spoken phonemes divided by the duration of voice-activated segments
*Acoustic features—Subset of eGeMAPS*^61^		
	F0semitoneFrom27.5Hz_sma3nz _meanRisingSlope	The mean rising slope of pitch (logarithmic F0 on a semitone frequency scale) smoothed over time
	loudness_sma3_percentile20.0	The 20th percentile loudness (perceived signal intensity)
	loudness_sma3_percentile50.0	The 50th percentile loudness (perceived signal intensity)
	loudness_sma3_percentile80.0	The 80th percentile loudness (perceived signal intensity)
	loudness_sma3_meanRisingSlope	The mean rising slope of loudness
	spectralFlux_sma3 _amean	Mean spectral flux (difference of the spectra of two consecutive frames)
	spectralFlux_sma3 _stddevNorm	Standard deviation of spectral flux
	mfcc1_sma3_stddevNorm	Standard deviation of Mel-Frequency Cepstral Coefficient 1
	mfcc3_sma3_amean	Mean of Mel-Frequency Cepstral Coefficient 3
	mfcc3_sma3_stddevNorm	Standard deviation of Mel-Frequency Cepstral Coefficient 3
	F1bandwidth_sma3nz_stddevNorm	Standard deviation of the bandwidth of Formant 1
	F1amplitudeLogRelF0_sma3nz_amean	Mean of the relative energy of Formant 1 relative to Pitch (*F*0) frequency
	alphaRatioV_sma3nz_stddevNorm	Standard deviation of the alpha ratio (ratio between energy high and low frequency bands) in voiced regions
	spectralFluxV_sma3nz_stddevNorm	Standard deviation of spectral flux in voiced regions
	mfcc1V_sma3nz_stddevNorm	Standard deviation of MFCC 1 in voiced regions
	alphaRatioUV_sma3nz_amean	Mean of the alpha ratio (ratio between energy high and low frequency bands) in unvoiced regions
	spectralFluxUV_sma3nz_amean	Mean of spectral flux in unvoiced regions
	loudnessPeaksPerSec	Number of loudness peaks per second
	VoicedSegmentsPerSec	Number of voiced segments per second
	MeanUnvoicedSegmentLength	The mean length of the unvoiced segments
	equivalentSoundLevel_dBp	Equivalent sound level (LEq)

We extracted 34 *linguistic features* used in our previous work^29^, where they proved useful in distinguishing spontaneous speech from AD patients and healthy controls. These features contain (a) syntactic information based on part of speech (POS) tagging and grammatical constituency parsing, (b) lexical information such as word and sentence lengths and measures of lexical richness and diversity, as well as c) measures of repetitiveness. Compared to Heitz et al.^29^, we removed features with a large absolute correlation (>0.85) to another feature, to remove redundancy and avoid numerical instabilities caused by multicollinearity. In addition, we followed Cho et al.^60^ by constructing five features quantifying the average word frequency, age of acquisition (AoA), word familiarity, semantic ambiguity, and concreteness of nouns in the transcripts.

Our *acoustic feature* included a set of hand-crafted features: We quantified pause-related speech characteristics using the WebRTC Voice Activity Detector, which segments each audio file into voice and pause segments. We excluded leading and trailing pause segments (i.e., silence at the start and end of each file), and calculated the overall *fraction of pause*, as well as the mean and standard deviation of pause durations. In addition, we implemented two related measures of speaking speed, inspired by Tóth et al.^31^: *speech rate* (the number of spoken phonemes divided by the total audio length) and *articulation rate* (the number of spoken phonemes divided by the duration of voice segments).

In addition, we extracted the extended Geneva Minimalistic Acoustic Parameter Set (eGeMAPS)^61^ using the openSMILE toolkit^62^. eGeMAPS is a set of 88 theory-driven acoustic descriptors of speech, including parameters relating to voice frequencies, energy, spectral parameters, and Mel-Frequency Cepstral Coefficients. For details on these features, we refer the reader to Eyben et al.^61^. To reduce the number of features, we sorted them by a measure of effect size (absolute correlation to the composite cognitive scores on the Development set, see *Experimental Setup* below) and kept only the top 30 features. Since some of these were highly correlated and thus mostly redundant, we further removed features such that absolute correlations were below 0.85. Our final selection of eGeMAPS features is included in Table 6, while all eGeMAPS features are listed in Supplementary Table 11.

We preprocessed all feature values for our analysis in two steps: (a) Since SVR (our main regression model) is sensitive to outliers, we removed extreme feature values and imputed them based on the remaining feature values. This preprocessing step mirrored the approach used for cognitive scores (cf. section “Data preprocessing”), with the important difference that feature statistics are computed using only training data, to avoid data leakage. (b) To harmonize feature units of measurement and facilitate model interpretability, we standardized all features by subtracting the training mean and dividing by the training standard deviation.

### Predicting cognitive performance from spontaneous speech features

The main analysis investigated whether features of spontaneous speech could predict individual cognitive performance using regression-based machine learning models. Using Python’s sklearn library (v1.4), we trained SVR models to predict cognitive scores across multiple domains. SVR extends the support vector machine (SVM) framework to regression, fitting hyperplanes in a high-dimensional, non-linear space to model complex relationships between input features and outcomes. Hyperparameter testing on our Development Set motivated the use of a radial basis function kernel and a regularization parameter of $C=0.5$). As a complementary approach, we also trained Random Forest Regression models—an ensemble method based on decision trees known for its flexibility and robustness—to validate the consistency of predictive performance across algorithms.

**Fig. 6:**
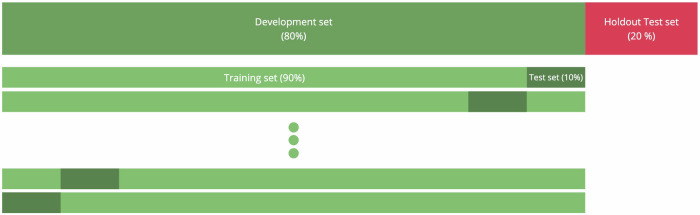
Experimental setup. The data were split into a Development set (green, 80% of the data = 788 participants) and a Holdout Test set (red, 20% of the data = 197 participants). On the Development set, 10-fold cross-validation was performed (light and dark green). CV results are reported on the union of the test sets (dark green). The Holdout Test set was used exclusively for final results.

We followed a rigorous experimental setup: To reserve a subset of the data for unbiased testing of our final models, we split the data into a Development set (80% of the data, corresponding to 788 participants) and a Holdout Test set (20% of the data, corresponding to 197 participants). Splitting was stratified for demographic information (age, country, gender, and education) and for the mean of the four composite cognitive scores. 18 participants had at least one missing variable required for stratification and were thus removed from the dataset. The Development set was used for model selection and hyperparameter tuning, and we evaluated models using stratified 10-fold cross-validation (CV). The independent Holdout Test set was used exclusively to test our final models, ensuring unbiased results. Figure 6 visualizes the experimental setup and data splits.

As a baseline setup, we first assessed the predictive power of demographic variables alone (age, gender, education, country), without including speech-derived features. A sensitivity analysis further evaluated the effect of including *socioeconomic status* as an additional demographic variable. In addition, we implemented two dummy baseline models: (a) a mean predictor, returning the mean training score for each test prediction and (b) a random predictor, which samples a score at random from the training set for each test prediction.

To evaluate the added value of speech-derived information, we then compared models incorporating acoustic features, linguistic features, or a combination of both, assessing the extent to which each feature set improved predictive model performance over the demographics-only baseline.

Models were evaluated using the *coefficient of determination* (*R*^2^), which is a popular global metric of model fit and can be interpreted as the amount of variance of the target variable explained by the independent variables^63^. We used the Python sklearn implementation, defined in Eq. (1), given *n* test participants with cognitive scores *c*_1_…*c*_*n*_, their mean $\overline{c}=\frac{1}{n}{\sum }_{i=1}^{n}{c}_{i}$, and model predictions ${\widehat{c}}_{1},\ldots {\widehat{c}}_{n}$.

1 $${R}^{2}=1-\frac{{\sum }_{i=1}^{n}{c}_{i}-{\widehat{c}}_{i}}{{\sum }_{i=1}^{n}{c}_{i}-\overline{c}}$$

Note that our dummy baseline algorithms have an expected value of *R*^2^ = 0 (for the mean predictor) and *R*^2^ = −1 (for the random predictor). To complement *R*^2^ scores, we calculated two additional evaluation metrics: (a) *Spearman correlation coefficient* (*ρ*) between *c*_1_…*c*_*n*_ and ${\widehat{c}}_{1},\ldots {\widehat{c}}_{n}$, which can be interpreted as the degree to which the model can distinguish between individuals with higher vs. lower cognitive performance. (b) *Mean absolute error* (${\rm{MAE}}=\frac{1}{n}{\sum }_{i=1}^{n}|{c}_{i}-{\hat{c}}_{i}|$), a popular metric for regression.

We estimated the variability of test results on the Development set using two-sided 95% bootstrap confidence intervals with 1000 bootstrap samples.

A statistical analysis was employed to test whether speech features improve predictive performance significantly compared to using demographics alone. To this end, we performed a non-parametric bootstrapping test: For each of 1000 bootstrap samples of the test participants, we computed the performance difference between all features and demographic features. Improvement was considered significant if 95% of these differences were larger than zero.

We examined which features have a large impact on model predictions using a feature importance analysis based on SHAP values^64^, a popular model-agnostic approach quantifying how much each feature contributes to a prediction. Furthermore, we evaluated the model performance across subgroups defined by age ranges, gender, country (USA vs. UK), and educational level to identify potential demographic model biases. For our main analysis, the two picture description tasks were concatenated into a single, extended speech sample per participant. In addition to this combined analysis, we also trained separate models on each individual picture description task and on the journaling task.

### Detection of cognitive low performers

Considering the number of included individuals and their age range, it is plausible that some participants were already experiencing early cognitive decline. To explore this further, we conducted a secondary analysis to evaluate whether spontaneous speech features could identify individuals whose cognitive performance was significantly below expectations based on their demographic information.

More precisely, these *cognitive low performers* were identified by establishing normative scores for each cognitive domain using the full dataset. We estimated linear norms of the four cognitive composite scores (*language*, *executive function*, *memory*, and *speed*), controlling for age, gender, education, and country. Participants with a *z*-score below −1.96 in a cognitive domain were classified as *cognitive low performers*, indicating significant negative deviation from the expected demographic norm.

We trained an SVM binary classification model in the same experimental setup as used in our regression analysis (cf. Fig. 6): We used the Development set for hyperparameter tuning and reported results using 10-fold cross-validation. In addition, we report results for the classification model trained on the Development set and tested on the Holdout Test set. We used two metrics of classification performance: the receiver operating characteristic (ROC) curve and the precision-recall (PR) curve, and the corresponding area under the curves (AUC). ROC–AUC is a popular and calibration-invariant global metric of classification performance. PR curves are recommended by many authors in the case of class imbalance, as they evaluate performance at different levels of recall. As a complementary approach, we also trained a Random Forest classification model to validate the consistency of predictive performance across algorithms.

### Evaluation on clinical dataset of AD patients and controls

In a third analysis, we aimed to evaluate the generalizability of our approach to clinical data. To this end, we tested whether the cognitive scores predicted by our primary regression models would be meaningful in an independent clinical AD population. While our primary dataset does not include AD patients, robust clinical validation is essential to demonstrate that the patterns captured by our models translate meaningfully to a distinct clinical cohort. We hypothesized that the predicted cognitive scores would be significantly lower for AD patients compared to healthy controls, reflecting known cognitive impairments typically associated with AD. To test this, we employed the ADReSS dataset^65^, a benchmark dataset for AD detection from spontaneous speech, consisting of 78 AD patients and 78 age- and gender-matched healthy controls. For each of the 156 participants, an audio recording of the Cookie Theft picture description task and the clinical diagnosis (AD or control) is available.

We preprocessed the audio files following the same pipeline as our main dataset, including automatic speech recognition and extraction of demographic, linguistic, and acoustic features (see sections “Data preprocessing” and “Speech feature extraction and preprocessing”).

Using the predictive models trained on the Development Set of our primary dataset, we generated predicted scores for the four cognitive domains (*language*, *executive function*, *memory*, and *speed*) based on each ADReSS participant’s picture description recording. Note that the ADReSS dataset differs from our study design, audio quality, and participant demographics. No ADReSS data was used for training or fine-tuning the employed prediction models. While classification performance would improve if we fine-tuned our model on the ADReSS dataset characteristics, we explicitly chose this setup to test the generalizability of our approach.

As ground truth cognitive scores are not available in ADReSS, we validated predictions by comparing the AD and control groups. We conducted independent-samples *t*-tests and calculated Cohen’s *d* for each predicted cognitive composite score.

## Supplementary information

Supplementary Information

## Data Availability

As voice recordings are considered personally identifiable information according to European legislation, the dataset generated during the present study cannot be deposited in a public repository in its entirety. However, a subset of participants (*n* = 837) have consented to the use of their data for further, non-commercial research. Cognitive test results and speech-derived feature values of these participants are published on GitHub (https://github.com/jheitz/speech_cognitive_biomarker), allowing replication and independent validation (Supplementary Table 12 presents results on this subset of participants). Moreover, upon reasonable request and in the context of a scientific collaboration, the authors can make the audio recordings and transcripts from this subset available to researchers. Our code for data preparation, feature extraction, modeling, and analysis is available on GitHub (https://github.com/jheitz/speech_cognitive_biomarker).
