# Supplementary material for: Towards a speech-based digital biomarker for cognitive impairment: speech as a proxy for cognitive assessment
Source: NPJ Digit Med. 2026 Jan 31;9:179. doi: 10.1038/s41746-026-02360-8 (PMC12917134; doi:10.1038/s41746-026-02360-8)
Supplement: Supplementary file 1 — Supplementary Information [file 41746_2026_2360_MOESM1_ESM.pdf]

Supplementary Material

Supplementary Table 1: Reliability results of the automatic scoring of standardized language tasks. We report intraclass correlation (ICC) as a measure of inter-rater reliability and mean absolute error, along with distributional statistics.

Task	Distribution of manual scores	ICC	Mean absolute error	Error distribution (Manual - Automatic)
Semantic fluency	Mean: 20.1, Std: 5.7, Min: 8, Max: 33	0.98 [0.97 0.99]	0.80	Mean: 0.60, Std: 1.1, Min: -2, Max: 4
Phonemic fluency	Mean: 16.4, Std: 5.2, Min: 4, Max: 26	0.98 [0.97 0.99]	0.85	Mean: 0.80, Std: 1.0, Min: -1, Max: 3
Boston Naming Test	Mean: 17.6, Std: 2.9, Min: 8, Max: 20	0.97 [0.94 0.98]	0.68	Mean: 0.68, Std: 0.8, Min: 0, Max: 3

Supplementary Table 2: Statistics for the original cognitive scores (ACS tests and standardized language tests). RAVLT: Rey Auditory Verbal Learning Test.

Test	Outcome measure	Mean	Std	Min	Max	Distribution
Trail Making Test A	Completion time [sec]	36.8	8.6	18.9	73.6	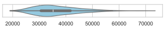
Trail Making Test B	Completion time [sec]	62.7	18.9	29.2	142.9	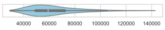
RAVLT (Learning)	Total number of correct words	50.3	11.4	13.0	75.0	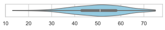
RAVLT (Recall)	Total number of correct words	10.2	3.4	0.0	15.0	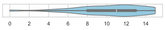
RAVLT (Recognition)	Total number of correct words	28.9	1.5	23.0	30.0	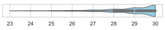
Visual Reaction Time	Mean reaction time [msec]	323.1	60.1	157.0	554.8	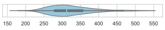
Corsi Block-tapping Test	Total number of correctly repeated sequences	9.2	1.6	3.0	15.0	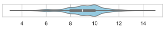
Grooved Pegboard	Completion time [sec]	67.1	14.3	40.9	122.3	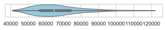
Digit Span (forward)	Total number of correctly repeated sequences	12.0	2.4	4.0	16.0	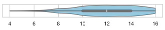
Digit Span (backward)	Total number of correctly repeated sequences	9.7	2.9	0.0	14.0	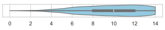
Clicking speed test	Completion time [sec]	25.7	7.7	10.9	56.3	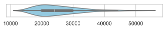
Mouse dragging speed test	Completion time [sec]	33.3	7.5	14.4	65.2	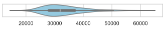
Phonemic Fluency ("F" fluency)	Total number of valid words	15.2	4.6	2.0	39.0	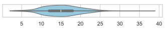
Semantic Fluency (Category fluency)	Total number of valid words	20.8	5.7	0.0	42.0	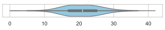
Boston Naming Test	Total number of correctly named objects	17.4	2.8	2.0	20.0	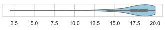

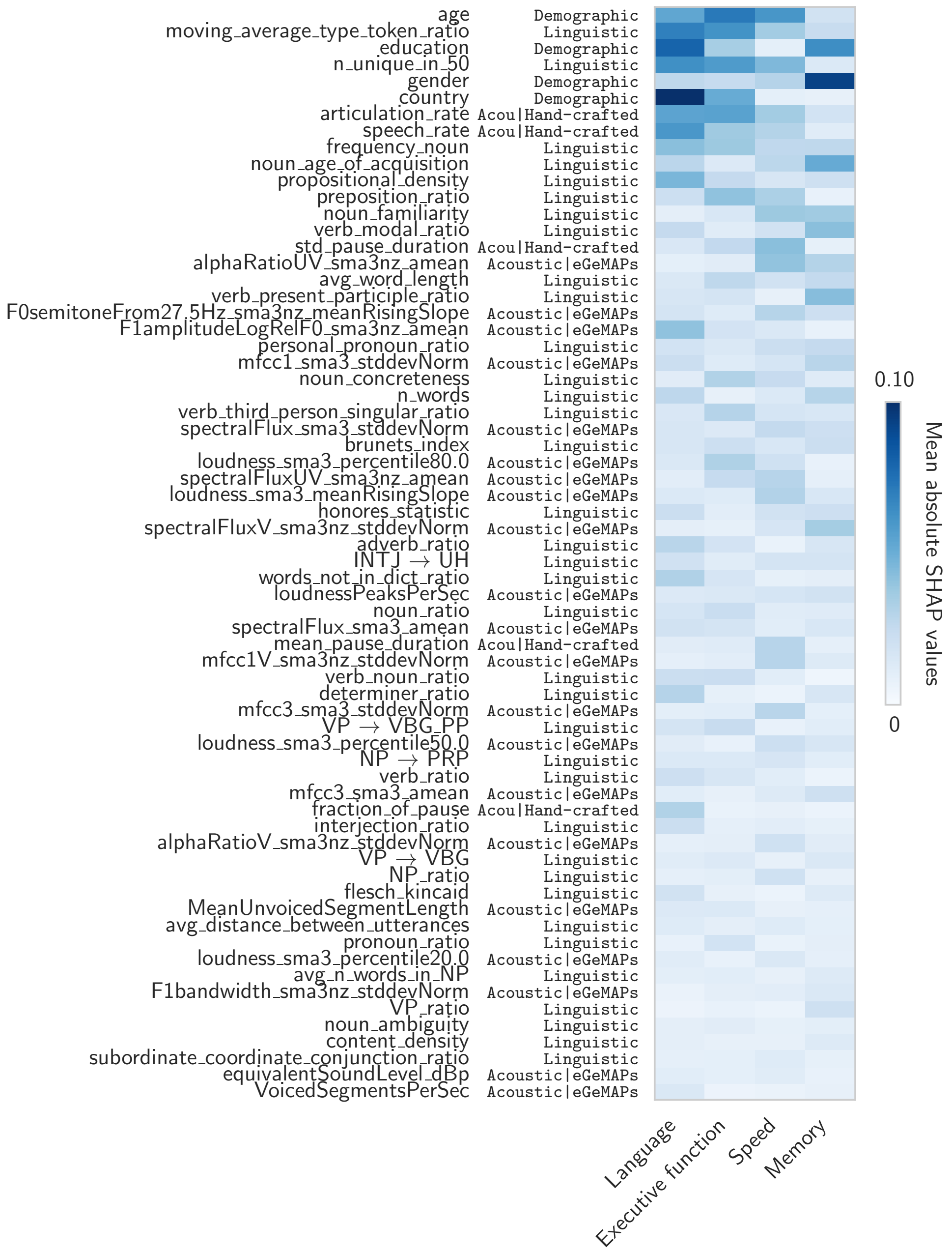

Supplementary Figure 1: Feature importance analysis of our main regression results for all four cognitive composite scores. This figure extends Figure 3 in the main results section, which focuses on the *language* score exclusively. Features are sorted according to the mean absolute SHAP value over the four target scores.

Supplementary Table 3: Various measures of model fit of the final confirmatory factor analysis (CFA) model for the first data half (used for iterative model improvement), second data half (used for validation of the improved model), and the entire data set (used for computation of cognitive composite scores).

	Robust CFI	Robust TLI	Robust RMSEA
First data half<br>(for model refinement)	0.948	0.931	0.055
Second data half<br>(for validation)	0.917	0.890	0.068
Entire data set<br>(for computation of cognitive composite scores)	0.945	0.927	0.057

Supplementary Table 4: Extension of our main results in Table 2, with additional metrics *Spearman correlation ρ* and *mean absolute error (MAE)*.

**Coefficient of determination** R^2 *(as reported in Table 2)*	Language		Executive function		Speed		Memory	
	CV Developm set	Holdout Test	CV Developm set	Holdout Test	CV Developm set	Holdout Test	CV Developm set	Holdout Test
Baseline: Random	-1.08 [-1.32, -0.87]	-0.81	-1.10 [-1.32, -0.90]	-1.30	-0.85 [-1.07, -0.69]	-1.03	-1.10 [-1.35, -0.91]	-1.01
Baseline: Mean predictor	-0.00 [-0.00, -0.00]	-0.00	-0.00 [-0.00, 0.00]	-0.03	-0.00 [-0.00, -0.00]	-0.00	-0.00 [-0.00, -0.00]	-0.01
Demographic features	0.07 [0.03, 0.10]	0.08	0.03 [-0.00, 0.07]	0.05	-0.00 [-0.04, 0.04]	-0.04	0.01 [-0.04, 0.05]	0.09
Audio+Demographic features	0.17 [0.12, 0.22]	0.17	0.08 [0.03, 0.12]	0.09	0.04 [-0.02, 0.08]	-0.01	0.01 [-0.04, 0.06]	0.03
Linguistic+Demographic features	0.23 [0.18, 0.28]	0.24	0.10 [0.05, 0.14]	0.18	-0.01 [-0.05, 0.03]	0.00	0.03 [-0.02, 0.09]	0.07
Linguistic+Audio+Demographic features	0.27 [0.22, 0.32]	0.29	0.13 [0.09, 0.18]	0.20	0.05 [0.01, 0.09]	0.03	0.04 [-0.00, 0.09]	0.07
**Spearman correlation** ρ	Language		Executive function		Speed		Memory	
	CV Developm set	Holdout Test	CV Developm set	Holdout Test	CV Developm set	Holdout Test	CV Developm set	Holdout Test
Baseline: Random	-0.01 [-0.10, 0.05]	0.09	-0.02 [-0.09, 0.05]	0.03	0.05 [-0.01, 0.12]	0.10	0.01 [-0.06, 0.08]	-0.01
Baseline: Mean predictor	-0.07 [-0.13, -0.00]	nan	-0.07 [-0.13, 0.01]	nan	-0.06 [-0.13, 0.01]	nan	-0.07 [-0.13, 0.00]	nan
Demographic features	0.25 [0.18, 0.31]	0.26	0.20 [0.14, 0.26]	0.24	0.13 [0.06, 0.20]	0.08	0.20 [0.13, 0.26]	0.38
Audio+Demographic features	0.39 [0.32, 0.44]	0.41	0.27 [0.20, 0.33]	0.34	0.27 [0.19, 0.32]	0.26	0.22 [0.16, 0.28]	0.28
Linguistic+Demographic features	0.45 [0.39, 0.50]	0.43	0.31 [0.25, 0.37]	0.42	0.17 [0.10, 0.22]	0.21	0.22 [0.15, 0.30]	0.31
Linguistic+Audio+Demographic features	0.50 [0.44, 0.55]	0.50	0.35 [0.29, 0.41]	0.45	0.27 [0.21, 0.33]	0.28	0.25 [0.18, 0.31]	0.29
**Mean absolute error** (MAE)	Language		Executive function		Speed		Memory	
	CV Developm set	Holdout Test	CV Developm set	Holdout Test	CV Developm set	Holdout Test	CV Developm set	Holdout Test
Baseline: Random	1.14 [1.08, 1.21]	1.03	1.16 [1.09, 1.22]	1.06	1.09 [1.02, 1.15]	1.06	1.13 [1.07, 1.19]	1.17
Baseline: Mean predictor	0.79 [0.75, 0.83]	0.79	0.81 [0.77, 0.86]	0.75	0.82 [0.77, 0.86]	0.74	0.81 [0.77, 0.85]	0.83
Demographic features	0.76 [0.72, 0.80]	0.76	0.79 [0.75, 0.84]	0.69	0.80 [0.76, 0.85]	0.76	0.78 [0.74, 0.83]	0.76
Audio+Demographic features	0.72 [0.68, 0.76]	0.70	0.78 [0.74, 0.83]	0.66	0.78 [0.74, 0.82]	0.72	0.78 [0.74, 0.83]	0.81
Linguistic+Demographic features	0.69 [0.66, 0.73]	0.69	0.76 [0.71, 0.80]	0.65	0.81 [0.76, 0.85]	0.72	0.78 [0.73, 0.82]	0.78
Linguistic+Audio+Demographic features	0.68 [0.64, 0.72]	0.66	0.75 [0.71, 0.79]	0.63	0.78 [0.74, 0.82]	0.70	0.77 [0.72, 0.81]	0.79

Supplementary Table 5: Results of our bootstrapped significance test. We report 95% confidence intervals for the R^2 performance difference between the use of exclusively demographic features compared to the use of the combined demographic, linguistic, and acoustic features. Results indicate a significant improvement for *language*, *executive function*, and *speed* scores.

Target variable	Performance increase (95% Confidence interval)	Significant finding
Language	0.15 - 0.24	True
Executive Function	0.05 - 0.14	True
Speed	0.01 - 0.10	True
Memory	-0.02 - 0.08	False

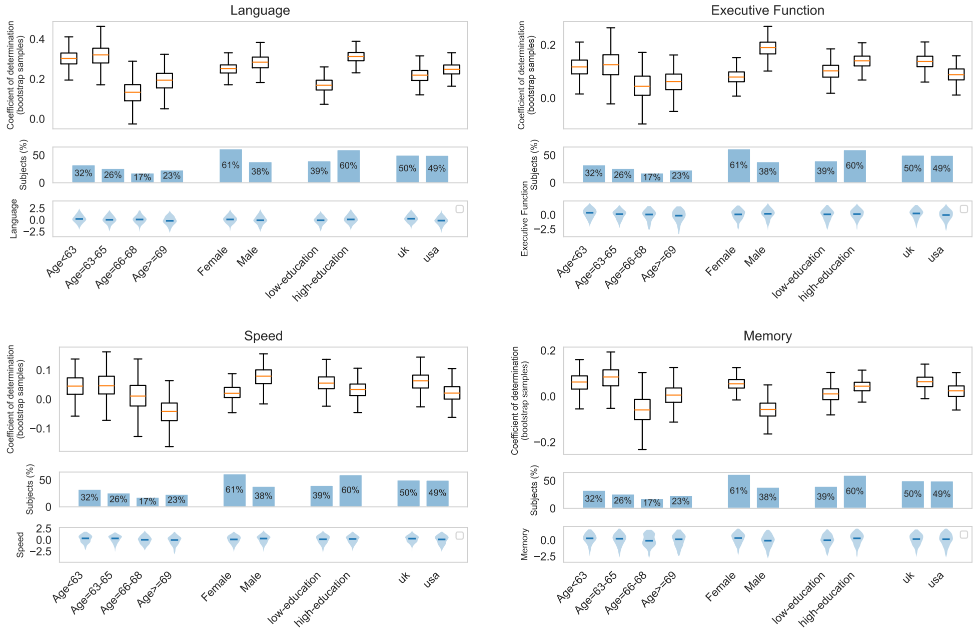

Supplementary Figure 2: Bias analysis, estimating regression performance (R^2) on different age ranges, genders, levels of education, and countries. Performance was estimated using bootstrap sampling. No large and consistent bias was found.

Supplementary Table 6: Regression results extracting features from a) the concatenation of both picture description tasks (our main analysis), b) the Cookie Theft picture description task, c) the Picnic Scene picture description task, d) the journaling spontaneous speech task.

**Coefficient of determination** R^2		Executive function		Language		Memory		Speed	
		CV Developm set	Holdout Test	CV Developm set	Holdout Test	CV Developm set	Holdout Test	CV Developm set	Holdout Test
	Demographic features	0.03 [-0.01, 0.07]	0.05	0.07 [0.04, 0.11]	0.08	0.01 [-0.04, 0.04]	0.09	-0.00 [-0.04, 0.04]	-0.04
Cookie Theft	Audio+Demographic features	0.07 [0.02, 0.11]	0.07	0.18 [0.13, 0.23]	0.12	0.00 [-0.06, 0.05]	0.05	0.01 [-0.03, 0.05]	-0.01
	Linguistic+Demographic features	0.08 [0.03, 0.12]	0.11	0.20 [0.15, 0.24]	0.17	0.05 [0.00, 0.09]	-0.00	-0.02 [-0.05, 0.02]	-0.03
	Linguistic+Audio+Demographic features	0.08 [0.04, 0.13]	0.19	0.23 [0.18, 0.28]	0.20	0.04 [-0.01, 0.09]	0.05	0.02 [-0.02, 0.06]	0.03
Picnic Scene	Audio+Demographic features	0.06 [0.02, 0.11]	0.11	0.15 [0.11, 0.20]	0.19	-0.00 [-0.05, 0.05]	0.03	0.02 [-0.03, 0.06]	0.02
	Linguistic+Demographic features	0.09 [0.05, 0.13]	0.14	0.22 [0.18, 0.28]	0.23	0.05 [0.00, 0.09]	0.08	-0.01 [-0.05, 0.03]	-0.00
	Linguistic+Audio+Demographic features	0.12 [0.08, 0.16]	0.19	0.26 [0.21, 0.30]	0.29	0.03 [-0.01, 0.08]	0.08	0.06 [0.02, 0.10]	0.04
Journaling	Audio+Demographic features	0.04 [-0.01, 0.08]	0.07	0.15 [0.09, 0.20]	0.16	0.02 [-0.04, 0.06]	0.00	0.01 [-0.03, 0.06]	-0.02
	Linguistic+Demographic features	0.09 [0.05, 0.13]	0.12	0.21 [0.16, 0.25]	0.15	0.02 [-0.03, 0.06]	-0.01	0.00 [-0.04, 0.04]	0.02
	Linguistic+Audio+Demographic features	0.10 [0.06, 0.14]	0.16	0.23 [0.18, 0.28]	0.22	0.02 [-0.03, 0.07]	0.02	0.05 [0.02, 0.09]	0.09
Picture Description	Audio+Demographic features	0.08 [0.03, 0.12]	0.09	0.17 [0.13, 0.23]	0.17	0.01 [-0.03, 0.06]	0.03	0.04 [-0.01, 0.08]	-0.01
	Linguistic+Demographic features	0.10 [0.05, 0.13]	0.18	0.23 [0.18, 0.28]	0.24	0.03 [-0.02, 0.08]	0.07	-0.01 [-0.05, 0.04]	0.00
	Linguistic+Audio+Demographic features	0.13 [0.09, 0.17]	0.20	0.27 [0.23, 0.32]	0.29	0.04 [0.00, 0.09]	0.07	0.05 [0.01, 0.09]	0.03

Supplementary Table 7: Regression results using an alternative regression model: Random Forest Regression.

**Coefficient of determination** R^2	Language		Executive function		Memory		Speed	
	CV Developm set	Holdout Test	CV Developm set	Holdout Test	CV Developm set	Holdout Test	CV Developm set	Holdout Test
Baseline: Random	-0.99 [-1.17, -0.81]	-0.94	-0.92 [-1.13, -0.72]	-1.20	-1.13 [-1.31, -0.94]	-0.95	-1.10 [-1.34, -0.90]	-0.98
Baseline: Mean predictor	-0.00 [-0.00, -0.00]	-0.00	-0.00 [-0.00, 0.00]	-0.03	-0.00 [-0.00, -0.00]	-0.01	-0.00 [-0.00, -0.00]	-0.00
Demographic features	-0.05 [-0.12, 0.02]	0.04	-0.04 [-0.11, 0.05]	-0.24	-0.10 [-0.17, -0.04]	0.04	-0.13 [-0.19, -0.06]	-0.21
Audio+Demographic features	0.15 [0.10, 0.21]	0.20	0.09 [0.04, 0.15]	0.06	0.04 [0.01, 0.09]	0.07	0.06 [-0.00, 0.11]	0.07
Linguistic+Demographic features	0.23 [0.18, 0.28]	0.21	0.10 [0.05, 0.15]	0.10	0.03 [-0.02, 0.08]	0.08	0.03 [-0.02, 0.07]	0.03
Linguistic+Audio+Demographic features	0.24 [0.18, 0.29]	0.26	0.13 [0.08, 0.18]	0.13	0.04 [0.00, 0.09]	0.05	0.08 [0.04, 0.13]	0.09

Supplementary Table 8: Sensitivity analysis of our main regression task, employing an *Extended* set of demographic variables, which includes *socioeconomic status (SES)* in addition to the *Default* set (age, gender, education, and country) as presented in Table 2. Results remain very similar.

**Coefficient of determination R^2**	Language		Executive function		Speed		Memory	
	CV Developm set	Holdout Test	CV Developm set	Holdout Test	CV Developm set	Holdout Test	CV Developm set	Holdout Test
Demographic features								
Default (age, gender, education, country)	0.07 [0.03, 0.10]	0.08	0.03 [-0.00, 0.07]	0.05	-0.00 [-0.04, 0.04]	-0.04	0.01 [-0.04, 0.05]	0.09
Extended (Default + SES)	0.07 [0.03, 0.11]	0.03	0.04 [-0.01, 0.07]	0.03	0.02 [-0.03, 0.06]	0.00	-0.00 [-0.05, 0.04]	0.07
Linguistic+Audio+Demographic features								
Default (age, gender, education, country)	0.28 [0.23, 0.32]	0.28	0.13 [0.08, 0.17]	0.20	0.05 [0.00, 0.09]	0.03	0.05 [-0.00, 0.09]	0.07
Extended (Default + SES)	0.28 [0.23, 0.32]	0.28	0.12 [0.08, 0.16]	0.20	0.05 [-0.00, 0.09]	0.04	0.05 [-0.00, 0.09]	0.07

Supplementary Table 9: Results of classification of cognitive low performers using an alternative Random Forest classification model

	# Samples		# Cognitive Low Performers		PR-AUC		ROC-AUC	
	CV Dev	Holdout Test	CV Dev	Holdout Test	CV Dev	Holdout Test	CV Dev	Holdout Test
Language	783	196	61 (7.8%)	8 (4.1%)	0.35 [0.23-0.50]	0.13	0.80 [0.74-0.86]	0.69
Executive Function	783	196	135 (17.2%)	26 (13.3%)	0.36 [0.28-0.44]	0.22	0.69 [0.64-0.74]	0.65
Speed	783	196	102 (13.0%)	23 (11.7%)	0.21 [0.15-0.28]	0.18	0.61 [0.56-0.67]	0.69
Memory	783	196	104 (13.3%)	33 (16.8%)	0.15 [0.12-0.21]	0.22	0.53 [0.48-0.59]	0.57

Supplementary Table 10: Number of outliers per ACS test and corresponding linear fit parameters. The test with the most outliers is Visual Reaction Time with 35 outliers, corresponding to 3.5% of all items. Outliers were then imputed using multivariate imputation.

		Linear Regression Fit Parameters				
Test	Number of Outliers	Age	Gender	Education	Country	Intercept
Trail Making Test A	11 (1.1%)	321.91	-26.72	-122.18	364.87	15583.27
Trail Making Test B	19 (1.9%)	733.15	-1878.96	-2915.83	4452.08	14992.57
RAVLT (Learning)	2 (0.2%)	-0.19	-4.47	3.79	-0.73	62.33
RAVLT (Recall)	0 (0.0%)	-0.03	-1.35	0.73	-0.12	12.18
RAVLT (Recognition)	9 (0.9%)	-0.02	-0.53	0.17	-0.27	30.58
Visual Reaction Time	35 (3.5%)	1.41	-14.29	1.49	13.74	228.61
Corsi Block-tapping Test	9 (0.9%)	-0.02	0.41	0.02	-0.25	10.77
Grooved Pegboard	26 (2.6%)	409.26	-81.48	108.71	1940.98	39260.93
Digit Span (forward)	2 (0.2%)	-0.04	0.30	0.31	0.24	13.92
Digit Span (backward)	0 (0.0%)	-0.03	0.27	0.22	0.06	11.67
Clicking speed test	21 (2.1%)	169.49	-1391.99	265.73	-526.46	15254.06
Mouse dragging speed test	21 (2.1%)	236.78	-1010.88	-837.90	1526.65	17921.73

```
(1) memory =~ ravlt_learning_correct_words + ravlt_recall_correct_words +
    ravlt_recognition_correct_words
(2) language =~ semantic_fluency_score + phonemic_fluency_score + picture_naming_score
(3) speed =~ mean_reaction_time + grooved_pegboard_total_time + clickskill_time +
    dragskill_time
(4) executive_function =~ trail_making_a_time_msec + trail_making_b_time_msec +
    digit_span_forward_correct_series + digit_span_backward_correct_series
(5) digit_span_forward_correct_series ~~ digit_span_backward_correct_series
(6) speed =~ trail_making_a_time_msec
```

Supplementary Figure 3: The `lavaan` model syntax of our final CFA model. Lines (1) to (4) result from the theory-driven assignment of test items to cognitive domains. The iterative model refinement based on modification indices (MI) resulted in two additional lines: (5) inclusion of the error covariance (MI 148.7), theoretically justified by a method effect, (6) inclusion of the cross-loading (MI 30.3), justified by the trail making test being strongly affected by speed of visual search.

Supplementary Table 11: All 90 eGeMAPS acoustic features, of which we removed 60 features due to low effect size on the Development set, and an additional 9 features due to high correlation with the remaining 21 features, which were kept for our final model.

Selected feature (21)	Removed due to low effect size (60)	Removed due to high correlation (9)
loudnessPeaksPerSec	F0semitoneFrom27.5Hz_sma3nz_stddevFallingSlope	loudness_sma3_stddevNorm
spectralFlux_sma3_stddevNorm	F2frequency_sma3nz_stddevNorm	F3amplitudeLogRelF0_sma3nz_amean
loudness_sma3_percentile50.0	dem_education_binary	F2amplitudeLogRelF0_sma3nz_amean
MeanUnvoicedSegmentLength	F0semitoneFrom27.5Hz_sma3nz_meanFallingSlope	StddevUnvoicedSegmentLength
spectralFlux_sma3_amean	hammarbergIndexV_sma3nz_stddevNorm	F1amplitudeLogRelF0_sma3nz_stddevNorm
VoicedSegmentsPerSec	mfcc2_sma3_amean	F3amplitudeLogRelF0_sma3nz_stddevNorm
spectralFluxUV_sma3nz_amean	F1bandwidth_sma3nz_amean	F2amplitudeLogRelF0_sma3nz_stddevNorm
F1amplitudeLogRelF0_sma3nz_amean	mfcc4_sma3_stddevNorm	loudness_sma3_amean
mfcc1V_sma3nz_stddevNorm	F1frequency_sma3nz_stddevNorm	spectralFluxV_sma3nz_amean
mfcc1_sma3_stddevNorm	F0semitoneFrom27.5Hz_sma3nz_amean	
loudness_sma3_percentile20.0	F3frequency_sma3nz_amean	
equivalentSoundLevel_dBp	F2bandwidth_sma3nz_amean	
F1bandwidth_sma3nz_stddevNorm	mfcc1V_sma3nz_amean	
F0semitoneFrom27.5Hz_sma3nz_meanRisingSlope	F0semitoneFrom27.5Hz_sma3nz_stddevRisingSlope	
mfcc3_sma3_amean	hammarbergIndexUV_sma3nz_amean	
spectralFluxV_sma3nz_stddevNorm	loudness_sma3_pctlrange0-2	
alphaRatioV_sma3nz_stddevNorm	F3bandwidth_sma3nz_amean	
alphaRatioUV_sma3nz_amean	F0semitoneFrom27.5Hz_sma3nz_stddevNorm	
loudness_sma3_meanRisingSlope	F0semitoneFrom27.5Hz_sma3nz_percentile20.0	
loudness_sma3_percentile80.0	F2frequency_sma3nz_amean	
mfcc3_sma3_stddevNorm	StddevVoicedSegmentLengthSec	
	dem_gender_unified	
	F0semitoneFrom27.5Hz_sma3nz_percentile50.0	
	mfcc4V_sma3nz_stddevNorm	
	F2bandwidth_sma3nz_stddevNorm	
	slopeUV0-500_sma3nz_amean	
	mfcc3V_sma3nz_amean	
	F0semitoneFrom27.5Hz_sma3nz_percentile80.0	
	F1frequency_sma3nz_amean	
	jitterLocal_sma3nz_amean	
	mfcc1_sma3_amean	
	loudness_sma3_meanFallingSlope	
	alphaRatioV_sma3nz_amean	
	mfcc2V_sma3nz_amean	
	F3frequency_sma3nz_stddevNorm	
	shimmerLocaldB_sma3nz_amean	
	shimmerLocaldB_sma3nz_stddevNorm	
	mfcc3V_sma3nz_stddevNorm	
	loudness_sma3_stddevRisingSlope	
	mfcc2V_sma3nz_stddevNorm	
	slopeV0-500_sma3nz_stddevNorm	
	slopeV500-1500_sma3nz_stddevNorm	
	slopeV0-500_sma3nz_amean	
	F3bandwidth_sma3nz_stddevNorm	
	logRelF0-H1-A3_sma3nz_stddevNorm	
	slopeUV500-1500_sma3nz_amean	
	hammarbergIndexV_sma3nz_amean	
	logRelF0-H1-H2_sma3nz_stddevNorm	
	logRelF0-H1-H2_sma3nz_amean	
	mfcc4_sma3_amean	
	slopeV500-1500_sma3nz_amean	
	HNRdBACF_sma3nz_amean	
	HNRdBACF_sma3nz_stddevNorm	
	logRelF0-H1-A3_sma3nz_amean	
	MeanVoicedSegmentLengthSec	
	loudness_sma3_stddevFallingSlope	
	F0semitoneFrom27.5Hz_sma3nz_pctlrange0-2	
	jitterLocal_sma3nz_stddevNorm	
	mfcc4V_sma3nz_amean	
	mfcc2_sma3_stddevNorm	

Supplementary Table 12: Regression results using an 84% subset of our data: It includes data on all participants who have consented to the use of their data for further, non-commercial research (*Development set*: 658 participants, *Holdout Test set*: 166 participants). This data can be shared upon reasonable request to the authors. The results are very similar to the results based on the entire dataset (Table 2), allowing independent reproduction and validation of our results.

**Coefficient of determination R^2**	Language		Executive function		Speed		Memory	
	CV Developm set	Holdout Test	CV Developm set	Holdout Test	CV Developm set	Holdout Test	CV Developm set	Holdout Test
Baseline: Random	-1.15 [-1.38, -0.92]	-1.02	-0.83 [-1.05, -0.63]	-1.37	-1.12 [-1.37, -0.92]	-0.79	-0.96 [-1.16, -0.76]	-1.06
Baseline: Mean predictor	-0.00 [-0.00, 0.00]	-0.00	-0.00 [-0.00, 0.00]	-0.02	-0.00 [-0.00, 0.00]	-0.00	-0.00 [-0.00, 0.00]	-0.02
Demographic features	0.07 [0.03, 0.11]	0.10	0.02 [-0.02, 0.05]	0.08	-0.01 [-0.05, 0.02]	0.02	0.00 [-0.04, 0.05]	0.07
Audio+Demographic features	0.16 [0.11, 0.22]	0.17	0.08 [0.03, 0.13]	0.10	0.03 [-0.02, 0.09]	-0.01	0.03 [-0.03, 0.08]	0.00
Linguistic+Demographic features	0.25 [0.19, 0.30]	0.26	0.10 [0.06, 0.15]	0.20	0.00 [-0.05, 0.04]	0.02	0.02 [-0.03, 0.08]	0.05
Linguistic+Audio+Demographic features	0.27 [0.22, 0.32]	0.28	0.13 [0.09, 0.18]	0.20	0.05 [0.00, 0.09]	0.04	0.05 [0.00, 0.10]	0.04

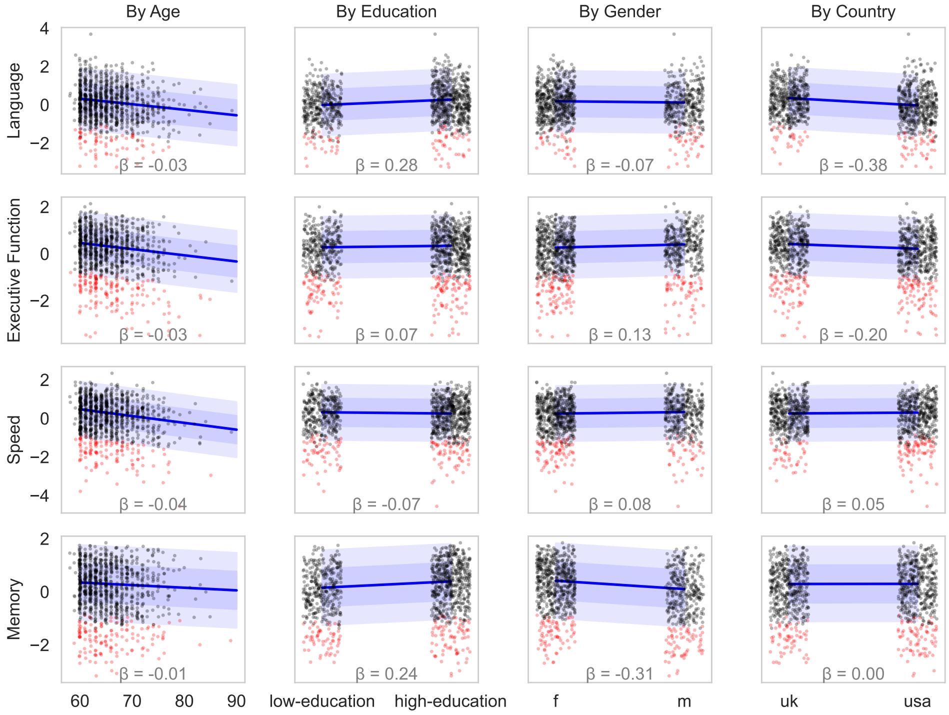

Supplementary Figure 4: Linear norms used to define *cognitive low performers* in our binary classification analysis. Norms are created independently for each cognitive domain (i.e. for each row). Blue lines depict the linear regression line (with beta coefficients in the lower center), shaded blue areas the standard deviation bands. Each data point in the dataset, including both Development and Holdout test set, is represented by a dot, with red dots representing the items identified as *cognitive low performers*. The number of *cognitive low performers* ranges between 69 (for *language*) and 161 (*executive function*) (Table 3).

Supplementary Table 13: Comparison of demographic variables and cognitive test results between UK and USA participants. For continuous variables, we report mean and standard deviation. p-values refer to a chi-squared test [1] (for categorical variables) or a Student's t-test [2] (for continuous variables). The observed differences between composite cognitive scores are the reason for the high feature importance scores for the demographic variable *country* observed in Figure 3.

	USA	UK	p-value
**Number of participants**	501	501	
**Age**	65.40 +- 4.69	65.53 +- 4.92	0.655
**Gender**			0.011
Female	327 (65.3%)	287 (57.3%)	
Male	174 (34.7%)	214 (42.7%)	
**Education**			< 0.001
High Education	334 (66.7%)	267 (53.3%)	
Low Education	164 (32.7%)	234 (46.7%)	
**Ethnicity**			< 0.001
White	433 (86.4%)	483 (96.4%)	
Black	45 (9.0%)	4 (0.8%)	
Mixed	14 (2.8%)	4 (0.8%)	
Other	6 (1.2%)	2 (0.4%)	
Asian	2 (0.4%)	6 (1.2%)	
**Socioeconomic status**	5.46 +- 1.75	5.71 +- 1.50	0.016
**Composite Cognitive Scores**			
Language	-0.19 +- 1.04	0.20 +- 0.92	< 0.001
Executive Function	-0.11 +- 1.01	0.11 +- 0.98	< 0.001
Memory	-0.02 +- 1.03	0.02 +- 0.97	0.631
Speed	-0.07 +- 1.05	0.07 +- 0.94	0.023
**Cognitive Test Scores**			
Trail Making Test A	37.03 +- 8.82	36.71 +- 8.39	0.562
Trail Making Test B	65.03 +- 18.58	60.90 +- 18.85	0.001
RAVLT (Learning)	50.34 +- 12.03	50.15 +- 10.71	0.793
RAVLT (Recall)	10.21 +- 3.37	10.12 +- 3.49	0.676
RAVLT (Recognition)	28.78 +- 1.55	28.98 +- 1.35	0.028
Visual Reaction Time	331.00 +- 63.23	316.16 +- 53.63	< 0.001
Corsi Block-tapping Test	9.05 +- 1.64	9.33 +- 1.60	0.006
Grooved Pegboard	68.54 +- 14.89	66.40 +- 13.93	0.019
Digit Span (forward)	12.16 +- 2.45	11.91 +- 2.39	0.097
Digit Span (backward)	9.76 +- 2.98	9.69 +- 2.89	0.705
Clicking speed test	25.58 +- 7.68	25.97 +- 7.68	0.414
Mouse dragging speed test	34.15 +- 7.98	32.71 +- 6.76	0.002
Phonemic Fluency ("F" fluency)	14.88 +- 4.60	15.60 +- 4.65	0.014
Semantic Fluency (Category fluency)	19.61 +- 5.58	21.92 +- 5.58	< 0.001
Boston Naming Test	17.06 +- 3.15	17.72 +- 2.23	< 0.001

Supplementary Table 14: Results of a linear model assessing the effect of the country of residence and covariates on the *language* composite cognitive scores. The country of residence remains a highly significant predictor, indicating that demographic differences alone cannot explain the group effects between the USA and UK displayed in Supplementary Table 13. *Note: Country*: 0=UK / 1=USA; *Education*: 0=low education / 1=high education; *Gender*: 0=female / 1=male; *Ethnicity*: 0=White / 1=Other. β = unstandardized regression coefficient; SE = Standard error; CI = Confidence interval.

	β	SE	CI	t-values	p-values
Intercept	-0.04	0.12	[-0.28, 0.20]	−0.33	0.742
Country	-0.37	0.06	[-0.50, -0.25]	−5.86	6.62e−09
Socioeconomic status	0.04	0.02	[-0.00, 0.08]	1.93	0.054
Education	0.25	0.07	[0.12, 0.38]	3.81	1.46e−04
Gender	-0.21	0.06	[-0.34, -0.09]	−3.29	0.001
Ethnicity	-0.45	0.36	[-1.15, 0.25]	−1.25	0.211

Supplementary Table 15: Reliability results of the automatic scoring of standardized language tasks per country. This table presents the information in Table 1 subdivided into countries.

Task	Country	True manual score	ICC	Error distribution (Manual - Automatic)	t-test for difference in errors
Semantic fluency	USA	Mean: 18.8, Std: 6.3	0.98 [0.94 0.99]	Mean: 0.65, Std: 1.4	t-stat: -0.29, p-value: 0.77
	UK	Mean: 21.5, Std: 4.7	0.99 [0.98 1.]	Mean: 0.55, Std: 0.7	
Phonemic fluency	USA	Mean: 16.1, Std: 5.2	0.99 [0.97 0.99]	Mean: 0.65, Std: 0.9	t-stat: 0.98, p-value: 0.33
	UK	Mean: 16.6, Std: 5.3	0.98 [0.96 0.99]	Mean: 0.95, Std: 1.0	
Boston Naming Test	USA	Mean: 16.8, Std: 3.6	0.99 [0.98 1.]	Mean: 0.50, Std: 0.5	t-stat: 1.47, p-value: 0.15
	UK	Mean: 18.4, Std: 1.5	0.85 [0.65 0.94]	Mean: 0.85, Std: 0.9	

References

[1] Kim, H.-Y.: Statistical notes for clinical researchers: Chi-squared test and fisher's exact test. Restorative dentistry & endodontics **42**(2), 152–155 (2017)

[2] Student: The probable error of a mean. Biometrika **6**(1), 1–25 (1908)
